# Supplementary material for: Case-control design identifies ecological drivers of endemic coral diseases
Source: Sci Rep. 2020 Feb 18;10:2831. doi: 10.1038/s41598-020-59688-8 (PMC7028714; doi:10.1038/s41598-020-59688-8)
Supplement: Supplementary file 1 — Supplementary information. [file 41598_2020_59688_MOESM1_ESM.pdf]

Case-control design identifies ecological drivers of endemic coral diseases - Supplemental Materials

Jamie M. Caldwell[1,2*], Greta Aeby[3], Scott F. Heron[2,4,5], and Megan J. Donahue[1]

1. Hawaii Institute of Marine Biology, University of Hawaii at Manoa, Hawaii, USA
2. ARC Centre of Excellence for Coral Reef Studies, James Cook University, Townsville, Australia
3. Department of Biological & Environmental Sciences, Qatar University, Doha, Qatar
4. Marine Geophysical Laboratory, Physics, College of Science and Engineering, James Cook University, Townsville, Australia
5. NOAA Coral Reef Watch, College Park, Maryland, USA

* contact: jamie.sziklay@gmail.com

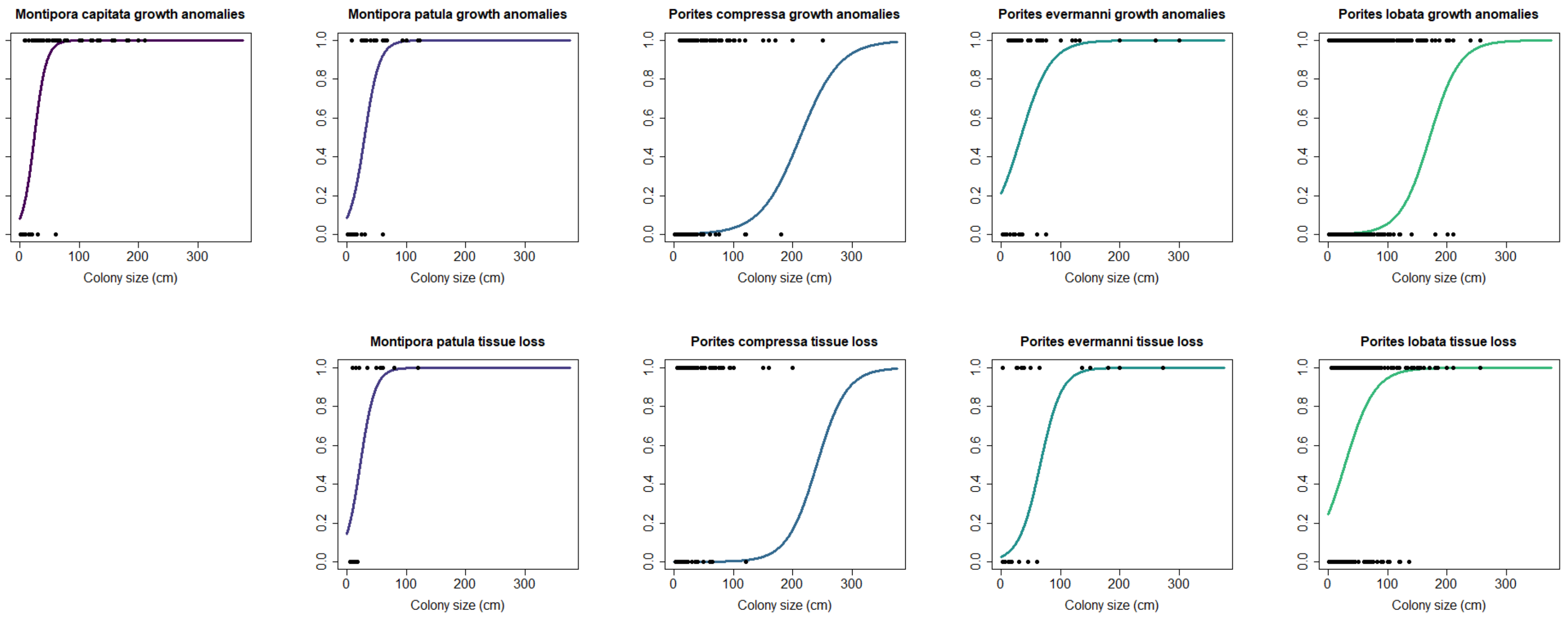

Figure S1: Disease risk as a function of colony size across host-disease pairs. Points indicate disease presence (P=1) and disease absence (P=0) from one random sample of a case control training dataset.

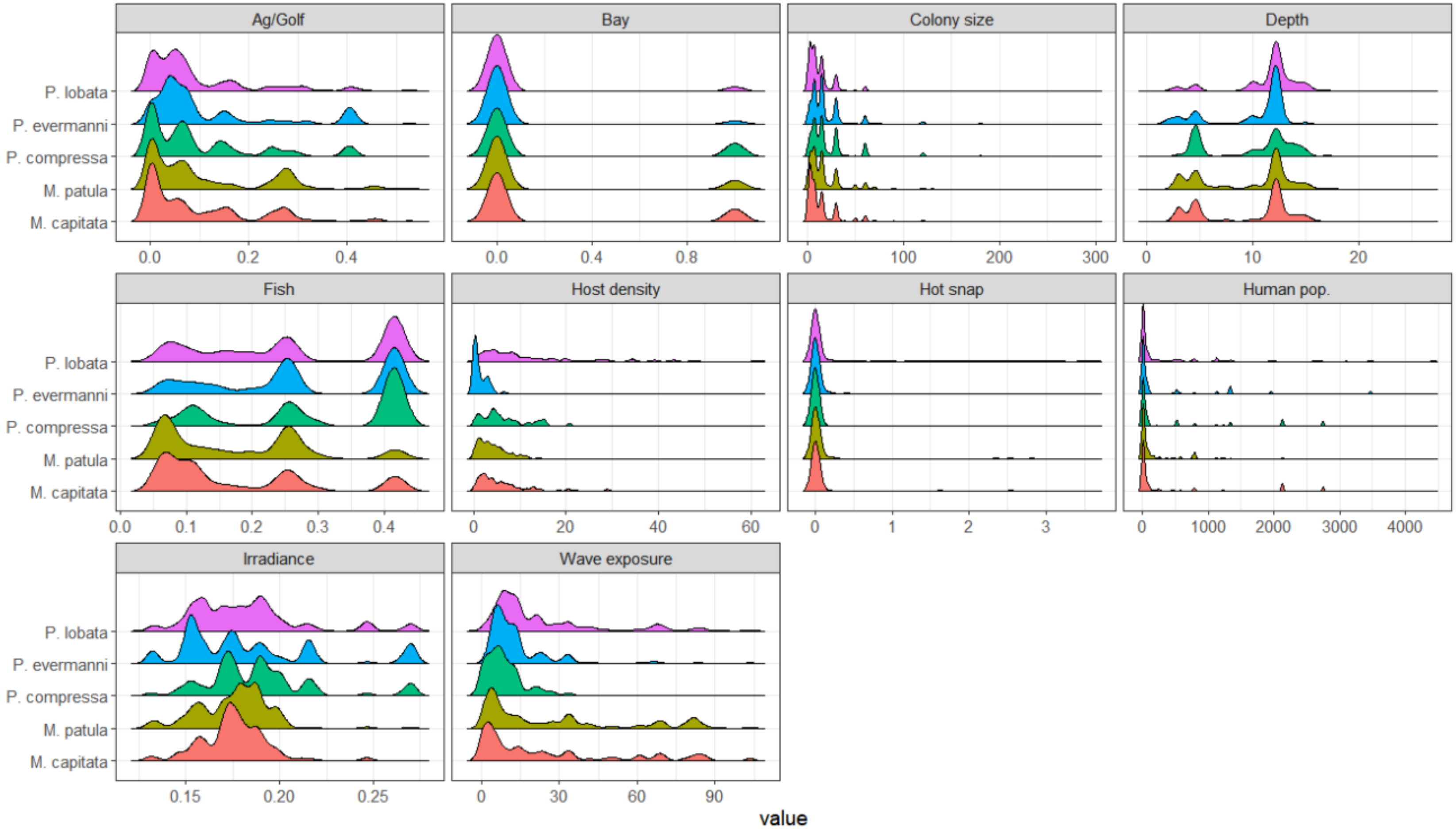

Figure S2: Distribution of risk factors (predictor variables) in growth anomaly models. Distribution of risk factors included in candidate growth anomaly models after removing observations with missing data. For the risk factor “Bay”, zero indicates outside a bay and one indicates inside a bay.

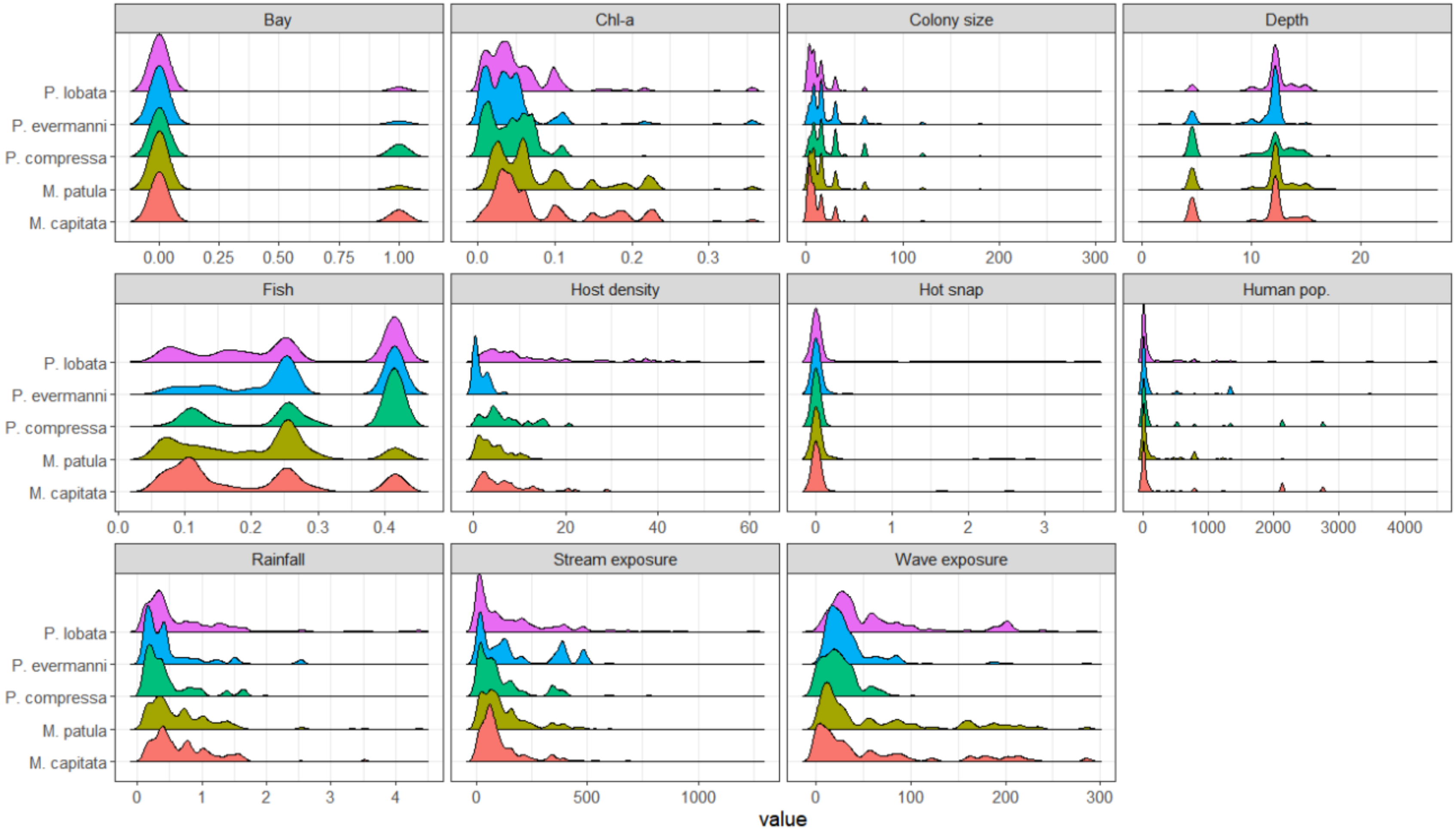

Figure S3: Distribution of risk factors (predictor variables) in tissue loss models. Distribution of risk factors included in candidate tissue loss models after removing observations with missing data. For the risk factor "Bay", zero indicates outside a bay and one indicates inside a bay.

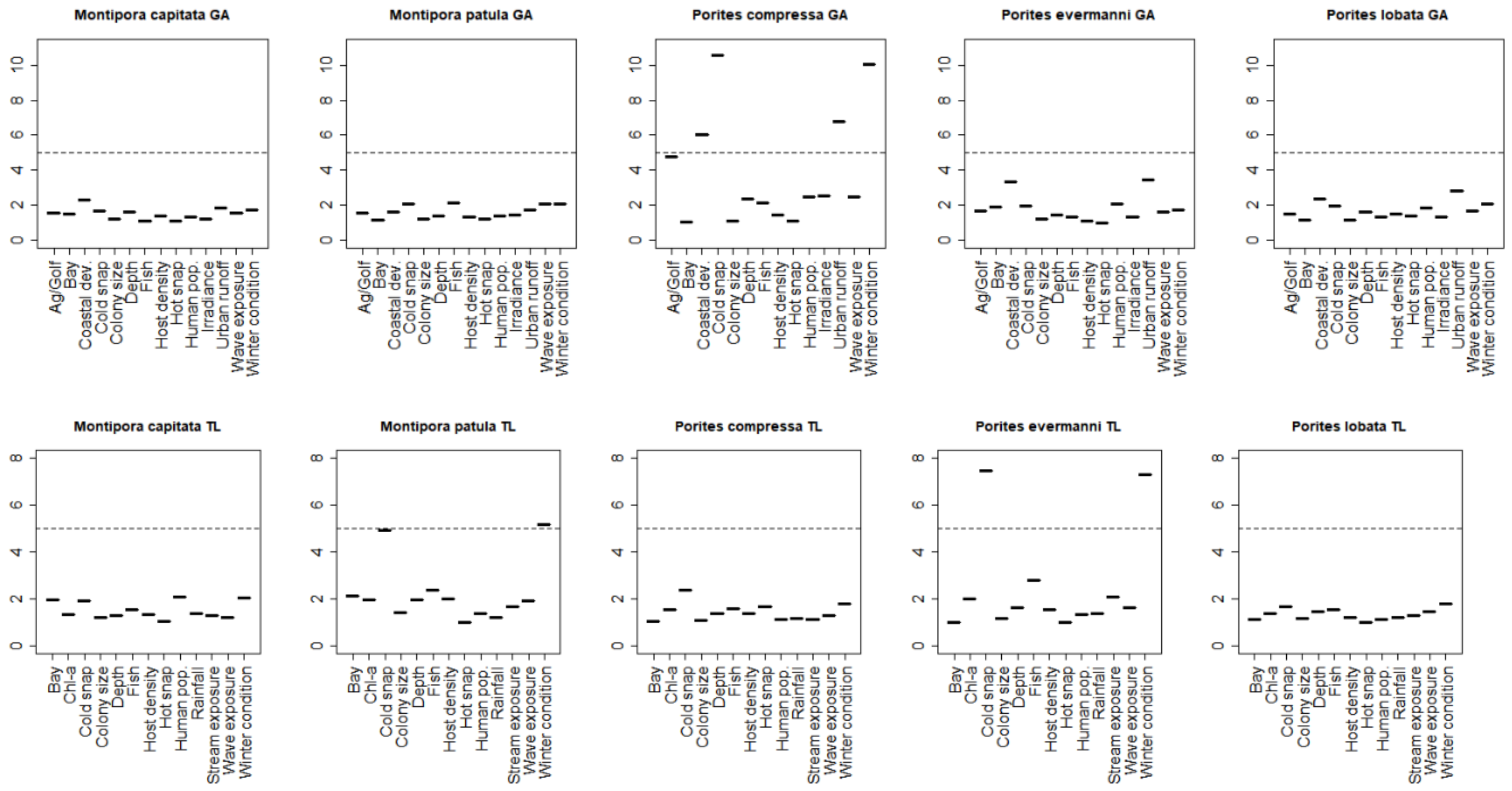

Figure S4: Variance inflation factor plots. Variance inflation factors for each predictor variable across host-disease pairs. Values greater than five (dashed horizontal line) indicate strong multicollinearity. For consistency, we removed predictor variables from all analyses if values exceeded five for any host-disease pair.

Table S1: Number of times the best fit model was selected as the best model within the 500 training datasets. Disease refers to growth anomalies (GA) and tissue loss (TL).

Disease	Species	Frequency of best fit model without case-control deign	Frequency of best fit model with case-control deign
GA	*Montipora capitata*	497	76
GA	*Montipora patula*	125	73
GA	*Montipora* spp	176	69
GA	*Porites compressa*	212	113
GA	*Porites evermanni*	111	36
GA	*Porites lobata*	278	87
GA	*Porites* spp	354	83
TL	*Montipora capitata*	187	23
TL	*Montipora patula*	92	43
TL	*Montipora* spp	154	47
TL	*Porites compressa*	125	86
TL	*Porites evermanni*	216	30
TL	*Porites lobata*	474	90
TL	*Porites* spp	139	63
